# Nutritional Status and Intra-household Food Distribution Among Reproductive-Age-Group Women in a Slum Area of Hooghly District, West Bengal: A Mixed-Methods Approach

**DOI:** 10.7759/cureus.24225

**Published:** 2022-04-17

**Authors:** Biswadip Chattopadhyay, Bobby Paul, Lina Bandyopadhyay, Madhumita Bhattacharyya

**Affiliations:** 1 Preventive and Social Medicine, All India Institute of Hygiene and Public Health, Kolkata, IND; 2 Occupational Health, All India Institute of Hygiene and Public Health, Kolkata, IND; 3 Maternal and Child Health, All India Institute of Hygiene and Public Health, Kolkata, IND

**Keywords:** malnutrition, nutritional status, women of reproductive age, mixed-methods study, gender bias, intra-household food distribution

## Abstract

Introduction

Malnutrition among women of reproductive age (WRA), especially those living in slum areas, is one of the most concerning nutritional issues because of the extreme nutritional stress they face in the form of inequitable intra-household food distribution (IHFD). This study aimed to assess the nutritional status (NS) and its association with IHFD among reproductive-age-group women along with exploring the perspectives of the stakeholders regarding inequitable food distribution.

Materials and methods

The quantitative part of the convergent parallel mixed-methods design study was conducted among 150 WRA, selected by cluster random sampling from 15 slum areas of Hooghly District, between December 2020 and May 2021. Data were collected using a predesigned pretested schedule with anthropometric measurements. IHFD was quantified by the relative dietary energy adequacy ratio (RDEAR). Ordinal logistic regression was performed to obtain adjusted-proportional odds ratios (aPOR) for higher categories of NS (underweight: reference category). Stratified subgroup analysis was done to assess the influencers of IHFD. For the qualitative part, in-depth interviews were conducted with eight purposively selected in-laws of study participants, and the data were interpreted by thematic analysis.

Results

The mean age of the study participants was 28.6±6.3 years. The proportion of malnutrition and inequitable IHFD (RDEAR<1) among them was 50% and 46%, respectively. Higher categories of NS were found to be significantly associated with an increase in RDEAR (aPOR=22.6, 95% CI: 2.75-185.45, p-value=0.004). Among underweight and normal NS women, those who were earning members and directly involved in food preparation/production had a greater allocation of food within their households. Physiological intolerance, incapacity of earning, and traditional customs were the most recurring themes transcribed as the barriers to equitable food distribution.

Conclusion

A high magnitude of malnutrition and its association with inequitable IHFD among WRA warrant policy-level support to increase women's employment opportunities and address gender-based inequities through comprehensive information education communication (IEC) techniques as well.

## Introduction

Malnutrition represents both under- and overnutrition and is a direct cause of varied complex health problems worldwide. Both of these could lead to the development of chronic diseases if not properly addressed [1]. According to the World Health Organization (WHO) classification, underweight and overweight/obese can be defined as a body mass index (BMI) of <18.5 kg/m^2^ and ≥25 kg/m^2^, respectively [2] Globally, around half of the adults are either underweight (12%) or overweight/obese (34%) [3]. Among them, due to certain hormonal and behavioral characteristics (like food deprivation in childhood and insufficient physical activities) women are at higher risk of developing malnutrition than men [4]; around 120 million women in the developing countries are underweight. Women of reproductive age (WRA), aged 15-49 years, are especially vulnerable to malnutrition; the prevalence of underweight and overweight/obese among Indian WRA was 21% and 23%, respectively, in 2016 [5]. This is worrying after accounting for the fact that more than half of the females in India are WRA, which represents around 250 million individuals. The nutritional status (NS) of WRA is indicative of the overall well-being of a population [6]. It has a crucial influence on the health of their own and that of the next generation. Since the last decade, India has been going through a rapid phase of urbanization, and around 34% of urban Indian women live in slums and are at the receiving end of extreme nutritional stress. Demographic health survey-4 in 2016 estimated that 20.6% of the urban poor women were thin/underweight and 21.1% of them were overweight/obese [7]. In this underprivileged section of urban society, a tangible gender-based disparity in nutritional aspects is displayed throughout the country. In India, the prevalence rate of malnutrition among ever-married women stands at 55.3% as against 24.2% among ever-married men [8]. Recent studies observed that the disparity is even more prominent among the tribal and slum population [8]. This huge gender gap points toward the lack of access to food for Indian women, which in turn points to inequitable intra-household food distribution (IHFD). A recent study in Indonesia has found a relationship between IHFD and dual forms of malnutrition (DFM) among adult women within the household [9]. Certain traditions, customs, and beliefs among especially vulnerable and socially marginalized communities, such as migrant and slum populations, magnify the inequity in food allocation within households. Although the WRA generally bear the brunt of this gender-based discrepancy in nutrition, there is a paucity of information pool within existing literature regarding the influence of IHFD on NS among WRA, especially in India.

In an attempt to dwell on the above-mentioned concerns, this study aimed to assess the level of NS among WRA in an urban slum area, and also the factors associated with it, with a special emphasis on IHFD. The study also aimed to explore the perspective of direct stakeholders regarding the inequity in household food distribution.

## Materials and methods

Study design, setting, and participants

This community-based mixed-methods study was conducted from December 2020 to May 2021 in the slum areas under Konnagar Municipality. The study design was convergent parallel (QUAN + qual); the quantitative strand was cross-sectional and the qualitative strand consisted of focus group discussions. A total of 19 slum areas were included in the study. Adult WRA (18-45 years) who were not pregnant or lactating (mothers with children less than one year) at the time of data collection were selected as study participants for the quantitative part. According to the USHA survey, SUDA-2019, the approximate number of WRA in the study area was 9540. No more than one WRA was selected from a single household. Women, in whose households there were no resident adult males eating household food, were excluded from the study. In-laws of the surveyed WRA were selected for in-depth interviews (IDIs), who have resided in the same household as the WRA at least for the past year.

Sample size determination

Cochran’s formula for determining sample size was applied for the quantitative part [10]. Standard normal variate was taken as 1.96 (5% type-I error), estimated proportion of malnutrition in WRA was taken as 0.38 as per National Family Health Survey 5 (NFHS-5) (West Bengal factsheet 2019-2020) on NS among that group [11], and the relative error in precision was taken as 25% in the study. After multiplying with a design effect of 1.5, the final estimated sample size came to 150.

The qualitative study sample size was determined as per the theory of data saturation.

Sampling design

Concurrent mixed-methods sampling (probabilistic sampling for quantitative strand and purposive sampling for quantitative strand) was implemented.

**Figure 1:**
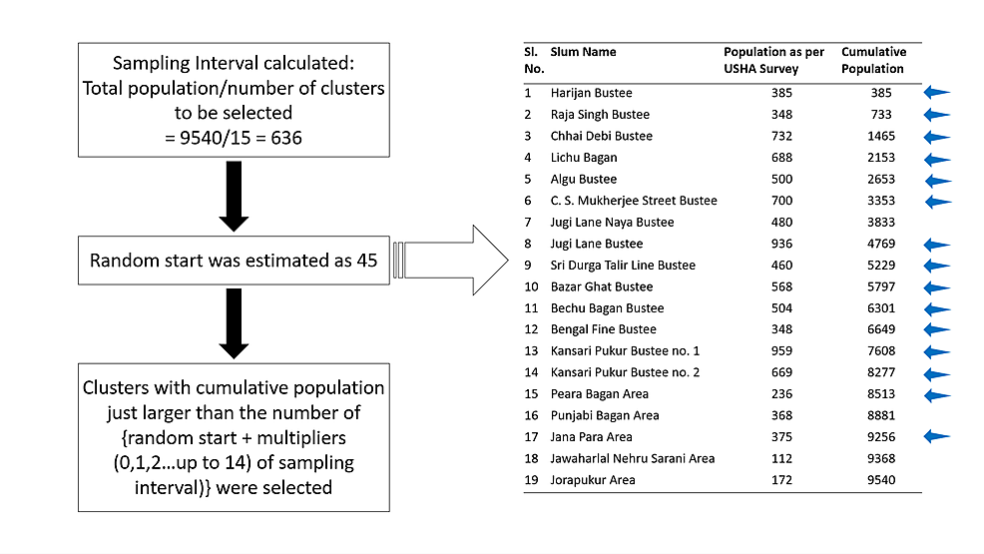
PPS method applied in the two-stage cluster sampling Blue arrow: clusters selected for the study. PPS, probability proportional to the population size.

A two-stage 15-cluster sampling technique was implemented with the help of the probability proportional to the population size (PPS) method. Each of the 19 slum areas was considered a cluster. In the first stage of the sampling, 15 clusters were selected after line-listing the slum areas according to population, with the help of a random start and sampling interval, as can be seen in Figure 1.

In the second stage, 10 households were selected from each of the 15 selected clusters (cluster size=sample size/number of clusters, i.e., 150/15=10) by simple random sampling technique after obtaining the household list from the respective honorary health workers assigned in that area. In case there was no eligible participant in a randomly selected household, the next household was selected.

Participants for the qualitative part were selected by a combination of different purposive sampling techniques, such as convenient, theoretical, and maximum variation sampling.

Data collection technique and tools

Quantitative data collection was done first followed by qualitative one. 

After building rapport with the study participants, face-to-face interviews were conducted using a pre-tested schedule. Local-language versions of the schedule were face and content validated by public health experts. Dietary assessment was done using the 24-hour recall method for the previous three days. Anthropometric measurement was done to assess NS. A calibrated digital weighing machine was used to measure body weight, while non-stretchable measuring tapes were used to assess height. Bodyweight was measured at three separate observations of 10-minute intervals during data collection and the average value was taken. Anemia status was assessed using a hemoglobin testing kit containing a digital hemoglobin monitor (Mission Hb ACON Biotech Co. Ltd.), test strips, puncturing lancet, micropipette, cotton swab, and spirit. IDI guides were used for conducting IDIs to the in-laws of study participants. They were instructed to come to the nearby subcenter on a predetermined date and time by local health workers. Study participants were initiated about the whole process and purpose of the study and included only after obtaining informed consent. The principles of public health research ethics were upheld during the study [12]. 

Study variables with operational definitions

NS among WRA (measured through BMI) was the study's dependent variable, categorized as per the WHO criteria [2]. The primary independent variable was IHFD, measured by the relative dietary energy adequacy ratio (RDEAR) [13]. RDEAR was denoted as the ratio of energy adequacy (average daily calorie intake/average daily calorie requirement) between a WRA and an adult male living and eating in the same household. The measurement of RDEAR included dietary assessment through the 24-hour recall method for three consecutive days and averaging it. The 24-hour recall method dietary assessment was only performed on weekdays (except on any holidays). Calorie requirements were estimated from the RDA report-2020 [14]. Any household with an RDEAR score of less than one was presumed to have inequitable food allocation to WRA. Dietary diversity was assessed by the Minimum Dietary Diversity for Women (MDD-W) Scale [15]. For each food group, food frequency was categorized as most of the days (daily to thrice-weekly), occasionally (twice a week-thrice a month), and rarely (<three times/month). Food insecurity of households was measured by Household Food Insecurity Access Scale (HFIAS) and the households were categorized into food secure, mildly food insecure, moderately food insecure, and severely food insecure [16]. Anemia severity categorization was done as per the WHO criteria [17]. The structured questionnaire also contained items assessing certain behavioral and lifestyle-related independent variables, such as perceived level of physical activity, the status of smoking and alcohol consumption, use of hormonal contraceptives, availability of improved drinking water, and sanitation facilities in the household. 

Statistical analysis

Quantitative and qualitative data analyses were done simultaneously. Quantitative analyses were performed on Microsoft Excel and Statistical Packages for Social Sciences (SPSS Inc. Released 2007. SPSS for Windows, Version 16.0. SPSS Inc., Chicago, IL). Proportions of NS and associated factors were represented through appropriate descriptive statistics. Chi-square tests were done to compare proportions of categorical independent variables among the response categories of NS (p<0.05 considered significant). Multivariable ordinal regression (with the help of a cumulative logit model) was performed to determine the association of different independent variables with response categories (underweight, normal, and overweight/obese, with underweight being the reference category) of NS. A generalized linear model was used to obtain adjusted proportional odds ratios. The selection of explanatory variables in the multivariable model was based on significance level in univariate analysis (p<0.05) and/or biological plausibility. The values of RDEAR among the sample were normally distributed as assessed by the normality statistics (Kolmogorov-Smirnov and Shapiro-Wilk tests), and subsequently, Student's t-tests were performed to compare mean values of RDEAR across categories of various factors in NS-stratified subgroup analysis.

Qualitative data were analyzed in an inductive approach; thematic analysis was done via manual coding.

## Results

Background characteristics

One hundred and fifty WRA were interviewed during the study with a mean age of 28.6±6.3 years. Out of them, the majority were Hindu (80.7%) and married (81.3%). More than half of the study participants (56%) were within the age bracket of 20-29. The educational level of the study subjects is varied with around one-fifth of the women have not had an education at the primary level, on the other hand about 40% had at least secondary or higher-level education. Around 58% of the WRA were homemakers, with around one-fourth of them being employed at the time of data collection. The background characteristics across different NS categories varied largely as can be seen in Table 1. 

**Table 1:** Background characteristics of the study participants across categories of nutritional status (N=150) *Column percentage backward classes=SC, ST, OBC; no anemia=blood Hb level >12.0 g/dL, mild anemia=Hb: 11-11.9 g/dL, moderate anemia=Hb: 8-10.9 g/dL, severe anemia=Hb <8 g/dL. ^#^Independent-sample Kruskal-Wallis test. **Fisher's exact test. Hb, hemoglobin.

Parameters	Variables		Overall no. (%)* (N=150)	Underweight no. (%) (n=26)	Normal no. (%) (n=75)	Overweight/obese no. (%) (n=49)	Test statistic; significance
Age in years: median (IQR)			28 (24-32)	26 (22.7-30)	28 (23-32)	28 (25.5-33.5)	2.15^#^; p-value: 0.34
Religion	Hindu		121 (80.7)	19 (15.7)	63 (52.1)	39 (32.2)	Chi-square: 1.53; p-value: 0.46
	Muslim		29 (19.3)	7 (24.1)	12 (41.4)	10 (34.5)	
Caste	Backward classes		45 (30)	12 (26.7)	20 (44.4)	13 (28.9)	Chi-square: 3.91; p-value: 0.14
	Others (general)		105 (70)	14 (13.3)	55 (52.4)	26 (34.3)	
Marital status	Married		122 (81.3)	22 (18)	59 (48.4)	41 (33.6)	Chi-square**: 0.63; p-value: 0.75
	Never married		28 (18.7)	4 (14.3)	16 (57.1)	8 (28.6)	
Educational level	Below primary		30 (20)	6 (20)	15 (50)	9 (30)	Chi-square: 9.29; p-value: 0.5
	Primary		31 (20.7)	9 (29)	16 (51.6)	6 (19.4)	
	Middle school		30 (20)	4 (13.3)	15 (50)	11 (36.7)	
	Secondary		26 (17.3)	2 (7.7)	11 (42.3)	13 (50)	
	Higher secondary		24 (16)	4 (16.7)	13 (54.2)	7 (29.2)	
	Graduate and above		9 (6)	1 (11.1)	5 (55.6)	3 (33.3)	
Employment	Unemployed/student		22 (14.6)	3 (13.6)	13 (59.1)	6 (27.3)	Chi-square: 1.17; p-value: 0.8
	Housewife		88 (58.7)	15 (17)	44 (50)	29 (33)	
	Employed		40 (26.7)	8 (20)	18 (45)	14 (35)	
Socioeconomic status (as per Modified BG Prasad Scale 2020)		Up to Class III	108 (72)	14 (13.0)	56 (51.9)	38 (35.1)	Chi-square: 5.26; p-value: 0.07
		Below Class III	42 (28)	12 (28.6)	19 (45.2)	11 (26.2)	
Anemia status		No anemia	81 (54)	6 (7.4)	40 (49.4)	35 (43.2)	Chi-square: 26.41; p-value: <0.001
		Mild anemia	22 (14.7)	3 (13.6)	12 (54.5)	7 (31.8)	
		Moderate anemia	43 (28.7)	14 (32.6)	22 (51.2)	7 (16.3)	
		Severe anemia	4 (2.6)	3 (75)	1 (25.0)	0	

Nutritional status and IHFD among the study participants

Out of the 150 women, 17.3% fell under the category of underweight, 27.3% overweight, and around 5% were obese. The median BMI of the study participants was 22.84 kg/m^2^. Half of the study participants were of normal NS. Approximately half of the households (46%) surveyed in the slum area suffered from inequitable food allocation to WRA (RDEAR<1). The mean RDEAR among the WRA in the study was 1.04±0.2 and the RDEAR values of the study participants were normally distributed. The differences of RDEAR mean values across response categories of NS (underweight: 0.91±0.17, normal: 1.04±0.19, overweight/obese: 1.12±0.2) were significant (analysis of variance [ANOVA]: F-statistics=9.67, p-value <0.001). A mild but significant positive correlation was established between the BMIs of the study participants and their respective RDEARs (Spearman’s rho: 0.381, p-value<0.001).

Independent variables for nutritional status

Around 90% of the study participants follow a non-vegetarian diet pattern, whereas only 52.7% have adequate dietary diversity as per MDD-W. As measured through HFIAS, more than half of the households are food secure, but about 20% of the households are moderately-to-severely food insecure. Only one-fifth of the women reported a strenuous perceived level of physical activities. The use of hormonal contraceptives was found among 28.7% of the study participants. In the case of the dietary pattern (frequency of consumption) for different food groups, the majority of the study participants consume cereals (77.3%), pulses (64.7%), meat-fish-egg (60%), and vegetables (56.7%) on most days, while eating dairy products (41.3%) and fruits (64%) rarely.

Ordinal regression analysis

A multiple ordinal regression analysis of NS among the study participants was conducted with underweight being the reference category for the dependent variable, as can be seen in Table 2. The cumulative logit model was acceptable to use due to the agreement on the proportional odds assumption (denoted by insignificant p-value =0.09). This means that the test of parallel lines null hypothesis could not be rejected, which states that the independent variable values are the same across response categories of the dependent variable. History of prior pregnancy, use of hormonal contraceptives, no anemia, frequent green vegetables, meat-fish-egg consumption, and increased RDEAR were found to be associated significantly with higher categories of NS. This particular model was fit to use due to the insignificant p-value (0.97) in Pearson goodness-of-fit test. As per the pseudo-R^2^ test, this model explained 40-49% of the variance of NS. The likelihood chi-square value in the Omnibus test found that the result of this model was significant (p<0.001).

**Table 2:** Multiple ordinal regression model of variables associated with higher categories of nutritional status ^#^Where nutritional status is of significant association with that respective independent variable. Dependent variable (nutritional status) categories for the ordinal regression model are as follows: Underweight (reference category), normal, and overweight/obesity.

Variables		Adjusted proportional odd’s ratio (95% confidence interval)	p-Value
Family type (reference: joint)	Nuclear	2.01 (0.89-4.57)	0.09
Socioeconomic status (reference: below Class III)	Up to Class III	1.25 (0.51-3.06)	0.62
Pregnancy status (reference: ever pregnant)	Never pregnant	0.17 (0.03-0.98)^#^	0.04
Use of hormonal contraceptives (reference: yes)	No	0.3 (0.12-0.74)^#^	0.01
Educational level (reference: above Class 8)	Up to Class 8	0.45 (0.18-1.13)	0.09
Anemia status (reference: anemia present)	No anemia	2.68 (1.2-6.0)^#^	0.01
Dietary habit (reference: other types of diet)	Non-vegetarian diet	0.18 (0.03-1.14)	0.07
Green vegetables eating frequency (reference: most days)	Rarely to never	0.2 (0.04-1.06)	0.05
	Occasionally	0.41 (0.18-0.92)^#^	0.03
Meat-fish-egg eating frequency (reference: most days)	Rarely to never	0.1 (0.02-0.49)^#^	0.005
RDEAR: intra-household food distribution ↑		22.6 (2.75-185.45)^#^	0.004

IHFD and associated factors

Certain social and economic factors were assumed to have a role to play in the distribution of food within a particular household. Three subgroup stratified analyses within each category of NS were done to compare mean values of RDEAR across categories of the factors, as can be seen in Table 3. Significant differences in mean RDEAR across any particular variables would be an indicator of its influence on IHFD. A relationship between IHFD was found with earning capacity, land ownership, and direct involvement of the WRA in household food production and preparation.

**Table 3:** Nutritional-status-stratified subgroup analyses comparing mean of RDEAR across categories of various factors among reproductive-age-group women (N=150) *Denotes statistically significant difference in RDEAR mean across respective categories (p<0.05 in Independent-sample t-test). RDEAR, relative dietary energy adequacy ratio.

Factors	Subgroup analysis (1): BMI<18.5 kg/m^2^ (n=26)		Subgroup analysis (2): BMI=18-24.9 kg/m^2^ (n=75)		Subgroup analysis (3): BMI≥25.0 kg/m^2^ (n=49)	
	RDEAR (mean±SD) among women with factors being present	RDEAR (mean±SD) among women with factors being absent	RDEAR (mean±SD) among women with factors being present	RDEAR (mean±SD) among women with factors being absent	RDEAR (mean±SD) among women with factors being present	RDEAR (mean±SD) among women with factors being absent
Earning member of the family	0.82±0.1*	0.96±0.17*	0.97±0.18*	1.06±0.19*	1.11±0.21	1.12±0.2
Decision-making in the procurement of food	0.93±0.18	0.86±0.08	1.06±0.17	1.02±0.2	1.11±0.21	1.13±0.19
Direct involvement in food production and preparation	0.86±0.09*	0.98±0.21*	1.02±0.17	1.08±0.23	1.11±0.21	1.15±0.17
Food-secure household	0.95±0.2	0.88±0.12	1.06±0.21	1.02±0.16	1.12±0.21	1.09±0.17
Joint family	0.94±0.19	0.87±0.09	1.05±0.21	1.02±0.16	1.11±0.21	1.12±0.19
Land ownership	0.96±0.21	0.87±0.06	1.06±0.18*	0.98±0.2*	1.16±0.2	1.1±0.2

Qualitative data findings

Eight participants (in-laws of the WRA) through the semi-structured IDI guide gave their perspective on IHFD, reasons for inequitable IHFD, and potential factors which could be beneficial to control the situation.

**Figure 2:**
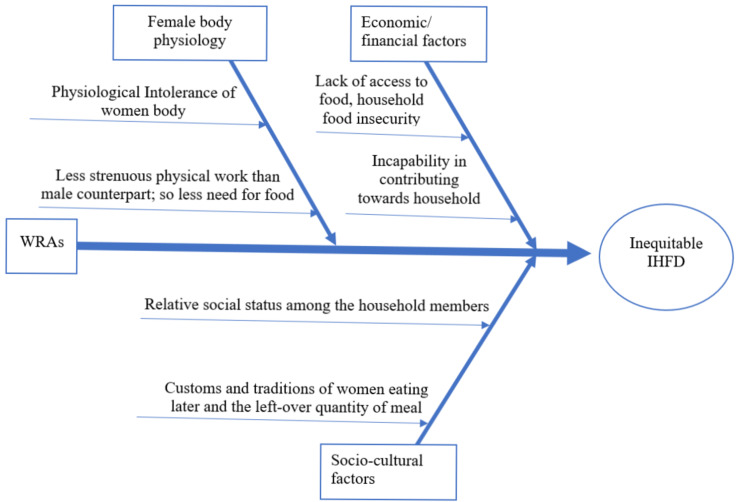
Ishikawa (cause-effect) diagram showing causes leading to inequitable IHFD among WRA as extracted through themes and subthemes from the qualitative analysis WRA, women of reproductive age; IHFD, intra-household food distribution.

The majority of the respondents (six out of eight) told that the physiological incapability to consumption of a large amount of food and lesser need for food due to comparatively less strenuous physical work were the main reasons for inequitable IHFD to WRA. Five out of the eight responders stated lack of financial contribution to the family and relatively lesser social status as the main barrier to equitable IHFD. Few of the respondents pointed out that most of the WRA, according to family custom and tradition, first serve the meals to the adult male of the house, who were generally the earning members of the family, which left very much less food for the women. A cause-effect diagram of the major themes and subthemes for barriers to equitable IHFD was constructed, as can be seen in Figure 2.

Although some of the respondents did not quote any occurrence of IHFD, the researcher observed an attitude of "getting a food share of your worth," whereas they indicated that if women had contributed more to the household/society, the incidence of inequitable IHFD would decrease. From the respondents’ perspective, improvement of housing conditions, women's employment, and campaign from health workers regarding nutritional health are some of the factors that could be beneficial for WRA in their allocation of food within the household.

Data triangulation

The themes that emerged from the qualitative findings were aligned with the adequacy status of IHFD, i.e., IDIs of in-laws who have had WRAs with RDEAR <1 reflected restrictive and overall negative themes, whereas in-laws who had WRAs with RDEAR of 1 or more in their household showcased positive viewpoint regarding the issue. A joint display was formulated to align the RDEAR status of WRAs with the major qualitative themes that emerged from their respective in-laws' illustrative quotes, as can be seen in Table 4. 

**Table 4:** Joint display of RDEAR findings and qualitative themes regarding food distribution KI, key informant; RDEAR, relative dietary energy adequacy ratio; IHFD, intra-household food distribution.

IHFD status	In-laws investigated for qualitative studies	Major qualitative themes	Quotable quotes
RDEAR<1 (inadequate IHFD)	5 (KI2, KI3, KI4, KI5, KI8)	Physiological incapability and lesser needs for food	KI3 quoted, “We try not to show any discrimination to our daughter-in-law, but as we all know that women are not capable of eating as much as a fully-grown man, that’s why they are given less food.”
		Lack of financial contribution to the household	KI2 quoted, “In my opinion, the men of the household are mainly given the majority of the food as they work hard for the bread-and-butter of the family.”
		Lack of access to food, household food insecurity	KI5 quoted, “We are having very little food in our house, which turned worse during the pandemic, shops are getting closed, the sellers are going to other places, and very few of them are coming in our slum due to fear of contracting coronavirus. As we are having food shortage, we tend to give the lion's share of our household food to our grandsons and males in the household.”
RDEAR≥1 (adequate IHFD)	3 (KI1, KI6, KI7)	Women empowerment (as a beneficial factor for equitable food distribution)	K1 quoted, “We share food equally amongst ourselves and it is not an issue with our family as we are somewhat in a better condition than most of our neighbors. My daughter-in-law works as a cook in different houses and gets meals there sometimes. But whenever she eats at home, we have equal food as she is also an integral part of the family. I think if women can be given jobs, they will also start to contribute to the procurement of food in the family.”
		Relative social status and custom	K7 quoted, “I think that women have less say in the matter of who is allocated how much of food. In our household, my daughter-in-law and I eat after the men as per our society’s custom, so sometimes we finish with less food eaten.”

## Discussion

This study assessed the status of malnutrition and its association with inequitable IHFD among WRA in a slum area. The high magnitude (50%) of DFM, i.e., the coexistence of both under- and overnutrition among the WRA in this study mirrors findings in research works from existing literature. A study from Bangladesh assessing DFM among WRA (non-pregnant, non-lactating) reported an age-adjusted prevalence of malnutrition as 41.8% [18]. As developing countries are going through a "nutrition transition," the prevalence of overweight/obesity is rapidly increasing and that of underweight continues to persist at the same level [19]. A larger magnitude of overnutrition (32.6%) than undernutrition (17.4%) in this study underlines the fact seen in recent trends, which reveal a shift of the burden of overweight from higher socioeconomic communities to relatively lower ones like tribal, urban poor, and slum areas, which might be explained by recent exposure to energy-dense foods, labor-saving devices, lack of concern/stigma about larger body sizes, sedentary occupations, etc. [20]. A study assessing the predicted burden of over- and underweight among WRA in several low- and middle-income countries, including India, highlighted that burden of DFM is becoming more and more prominent due to the rapid increase of overweight, especially in WRA belonging to the poorest wealth quintiles [21].

A longitudinal study by Song et al. found marital status, increased age, smoking history, and urban residence to be significantly associated with higher categories of NS among WRA [22]. A recent Indian study using WRA data from NFHS-4 had found associated factors for higher categories of NS similar to the present study [23]. A study in Ethiopia identified lesser consumption of fish and dairy products and food insecurity as independent predictors for malnutrition among WRA [24]. A study in Indonesia reported a significant positive association of DFM with IHFD carbohydrate but not with IHFD energy (which was measured by RDEAR in this study) among mothers [9]. This observation could be because consumption of micronutrients was scarce across different categories of weight. A study in Nepal found that women who earn similar or more than their respective spouses have significantly higher RDEAR than the rest [25]. A systematic review by Harris-Fry et al., about determinants of IHFD among adult women in South Asia, revealed few individual-level (such as income, bargaining power, social status, tastes and preferences, interpersonal relationships, etc.) and household-level factors (wealth, household food insecurity, household size, land ownership, etc.), some of which were also significantly associated with IHFD in the present study [26].

Qualitative analysis in the present study established the theme of physiological disparity as a barrier to equitable IHFD, as perceived by the in-laws of WRA. A similar hypothesis can be resonated in a qualitative study, where the researcher found that greater allocation of food to the males of the household was done based on a more strenuous physical workload compared to WRA [27]. Traditional customs pose a barrier toward equitable IHFD of WRA by inculcating beliefs that women are below men in relative social status or contribute less to the household or community, which generates the tendency to allocate less and less preferred food to the adult women of the household. A study by Coffey et al. also suggested relative social status as a determinant of food allocation within the household [28]. Also, it is common practice in Indian households for the women to eat after the adult male of the house, thus getting less preferred foods [29]. These pieces of information warrant discussions on the sociocultural construction of nutritional needs in India.

This study complements the evidence present in the existing literature on DFM and its association with inequities of IHFD. Along with finding out the association of IHFD with the nutritional status of WRAs, the study also triangulated relevant information regarding the socioeconomic and sociocultural construct of gender-based inequities in household food allocation that emerged from both quantitative and qualitative analyses. The present study also had a few limitations. First, the sample size was small and the study subjects were selected from relatively closely situated clusters which might affect the predictors of NS. To calculate RDEAR, any adult male residing in the household from a similar age bracket was included, with no specification on the relationship to the study participants, which might also affect the IHFD. Also, this study could not attribute causality to the association of IHFD to the social determinants. The effect of seasonality on IHFD could not be excluded. Qualitative validity was affected by limited field time due to the pandemic.

## Conclusions

This study found a positive association between undernutrition and inequitable IHFD among WRA. The results indicated that women’s NS would be bettered by an improvement in their IHFD patterns. Policymakers could implement women’s empowerment measures to reduce inequitable IHFD, like interventions that would provide earning opportunities to WRA, creating or advancing women’s self-help groups to provide livelihood opportunities and increase social mobility. Nutritional knowledge of WRA should be increased through comprehensive information education communication (IEC) activities which could drastically curb the prevalence of both malnutrition and inequitable IHFD among them. Selectively focused, target-oriented, evidence-based approaches should be implemented to change the perspective of direct stakeholders on the cultural norms that enable nutritional gender bias. To fully understand the extent of malnutrition and IHFD among WRA, more longitudinal mixed-methods research works should be done in different socioeconomic strata, regions, and seasons. No stones should be left unturned in the research of the important but neglected topic of IHFD.
